# Clinical presentation, histology, and prognoses of malignant melanoma in ethnic Chinese: A study of 522 consecutive cases

**DOI:** 10.1186/1471-2407-11-85

**Published:** 2011-02-25

**Authors:** Zhihong Chi, Siming Li, Xinan Sheng, Lu Si, Chuanliang Cui, Mei Han, Jun Guo

**Affiliations:** 1 Key laboratory of Carcinogenesis and Translational Research (Ministry of Education), Department of Renal Cancer and Melanoma, Peking University School of Oncology, Beijing Cancer Hospital & Institute, Beijing, China

## Abstract

**Background:**

Malignant melanoma is a rare disease in Asia, and knowledge on its characteristics and clinical outcome in Asian patients is limited. The purpose of this observational study was to determine the clinical presentation and outcome of patients with melanoma in China.

**Methods:**

A database was prospectively established for the purpose of this analysis. The elements of the database included basic demographic data of patients and prognosticators previously reported in literature, as well as follow-up data including clinical outcome after treatment. Medical record of all patients with pathologically diagnosed malignant melanoma consulted in our center since 2006 were retrieved and reviewed. No patient was excluded in this study. Statistical analyses including survival and multivariate analyses of factors associated with survival were respectively performed by Kaplan-Meier method and Cox proportional hazard model.

**Results:**

A total of 522 consecutive and nonselected cases were evaluated. There were 218 cases (41.8%) of acral lentiginous melanoma (ALM), 118 (22.6%) of mucosal melanoma (MCM), 103 (19.7%) of nodular melanoma (NM), 33 (6.3%) of superficial spreading melanoma (SSM), and others were Lentigo maligna melanoma or unclassifiable disease. The proportion of patients with clinical stage I, II, III, and IV diseases were 6.1%, 55.9%, 25.1%, and 12.8%, respectively. Among the 357 cases of cutaneous melanoma, 234 patients (65.5%) had ulceration.

The 5-year overall survival rate of all 522 patients was 41.6%, and the median survival time was 3.92 years (95% CI, 3.282 to 4.558). Five-year survival rates of patients with stage I, II, III, and IV diseases were 94.1%, 44.0%, 38.4% and 4.6% respectively (P < 0.001). Multivariate analysis revealed that clinical stage and the ulceration were two significant prognosticators for OS. In addition, extent of surgery and use of adjuvant therapy were significant prognosticators for DFS in patients with non-metastatic disease after definitive treatment. Pathological subtype was not a significant prognostic factor to predict wither OS or DFS.

**Conclusions:**

Prognoses of patients with malignant melanoma diagnosed in China were suboptimal, and most patients were diagnosed with locally advanced disease (i.e., stage II or above). ALM and MCM are the two most commonly diagnosed pathological subtypes. Clinical staging and presence of ulceration was significantly associated with clinical outcome in terms of OS, while treatment strategy including extent of surgery and use of adjuvant therapy were significant predictors of DFS.

## Background

Malignant melanoma demonstrates a clear demographic and ethnic disparity, and is a common malignancy in Western countries among people with light-colored skin. It has the highest incidence in Queensland, Australia [1], and is the 5th most commonly diagnosed cancer in the United States [2]. However, the malignancy is relatively rare among Africans, Hispanics, and Asians. In addition to the variations demonstrated in incidences, the Surveillance, Epidemiology, and End Results (SEER) data from the United States showed that the clinical characteristics such as pathology, anatomical origin, and patients' prognoses differ significantly among different ethnic groups [3]. Furthermore, although darker-pigmented populations including Asians may benefit from protective effect of melanin to ultraviolet radiation, worse prognoses for non-Caucasian melanoma patients including a significantly shorter survival time have been reported [4-6].

Based on the published literatures and reported disparities, it is reasonable to postulate that malignant melanoma diagnosed in non-Caucasian population differs from those diagnosed in endemic regions. However, despite the extensively published results from Western countries, knowledge of melanoma in Asian patients is scant. Current available literatures for the disease in Asia are limited to a survey, a small retrospective series, as well as an epidemiology report from U.S. that included Asian patients as a minority group [3,7-9]. Clinical evidence for Asian patients especially of large-scale does not exist due to, at least in part, the rarity of the disease in this region. As such, the etiology, characteristics, biological behavior, as well as outcome after treatment are largely unknown for melanoma in Asian patients. Clearly, additional knowledge on the characteristics of the disease as well as the outcome after treatment is needed to permit a better understanding of this highly aggressive and racial specific malignancy.

The aim of this analysis is to bolster the existing but highly limited literatures on malignant melanoma of Asian people by documenting the clinical presentation as well as outcome after active treatment of a relatively large group of Chinese patients with pathologically confirmed malignant melanoma recently treated in our tertiary referral center specialized for the management of this disease.

## Methods

### Database Design

A database was prospectively designed for the current analyses after the approval by the institutional review board (IRB) of the Beijing Cancer Hospital (BCH) (Beijing, China) prior to the retrieval and reviewing of medical records. Elements of the database were based on published prognostic factors in addition to basic demographic data, and included characteristics of the patients (age at diagnosis, gender, and performance status), disease (anatomic location, histological subtype, presence of ulceration, Breslow thickness, and stage), treatment (modality of treatment, type of surgery and systemic treatment agents used), and follow-up (time of disease recurrence/progression, patients' death, interval of local control and disease-free survival).

After the designing of the database, medical records of all patients presented to the Department of Renal Cancer and Melanoma of BCH with pathologically confirmed malignant melanoma were retrieved, reviewed, and accrued into the database.

### Patients and Staging Evaluation

According to the institutional protocol, pretreatment evaluation of all patients consisted of a complete history and physical examination, biopsy of the primary or secondary lesion with pathology study, complete blood count, serum chemistry, metabolic and liver panels, thoracic CT scan, and abdominal CT or ultrasound. Local excision is performed in patients with clinical stage I cutaneous melanoma without biopsy. Additional evaluation tests were required for patients who were accrued into our institutional prospective trials.

The American Joint Committee on Cancer (AJCC) staging system (6^th ^edition) was used for either clinical or pathological staging [10]. For externally referred patients without pathological evaluation of the Breslow thickness, clinical stages were determined based on the available T-category, regional lymph node involvement, as well as status of distant metastasis.

### Data Analysis

The duration of time to locoregional failure or distant metastasis was measured from the end of treatment for non-metastatic diseases until documented treatment failure. The duration of progression-free survival (PFS) in stage IV patients were from the initiation of any treatment (either systemic or local palliation) until documented disease progression at any site. The duration of overall survival (OS) was calculated from the pathologic diagnosis of melanoma until death or until the date of the last follow-up visit for patients still alive.

The actuarial local control, disease-free survival (DFS), PFS, and overall survival rates were calculated by Kaplan-Meier method [11]. Mantel-Cox log-rank test stratified by every factor was applied to compare the Kaplan-Meier curves for survival. Multivariate analyses of prognosticators for OS, DFS, and PFS were performed using Cox proportional hazard model. Variables with a P < 0.10 in univariate analyses were included in multivariate analysis.

## Results

Between January 2006 and March 2010, a total of 522 consecutive and non-selected cases of malignant melanoma with pathologic confirmation were identified and reviewed. No patient was excluded in this analysis. The median follow-up time for the entire group of patients was 16 months (range 1-87 months).

**Figure 1:**
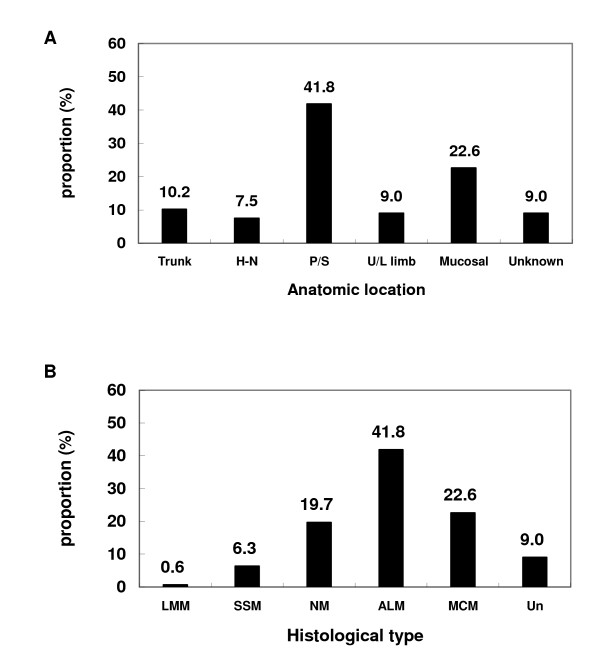
**Patient distribution by (A) anatomic location (H-N, head and neck) and (B) histological type (Un, unclassifiable)**.

Among the 357 cases of cutaneous melanoma including acral lentiginous melanoma (ALM), Superficial Spreading Melanoma (SSM), nodular melanoma (NM), and Lentigo Maligna Melanoma (LMM), 234 (65.5%) patients had ulceration in their primary lesion. Characteristics of the patients and their diseases are detailed in Table 1 and Figure 1.

**Table 1:** Characteristics of the patients (at diagnosis) and their diseases

Characteristics	No.	%
Age at diagnosis (years)		
≤ 65	429	82.2
> 65	93	17.8
**Gender**		
**Male**	**276**	**52.9**
**Female**	**246**	**47.1**
Anatomic location		
Trunk	53	10.2
Head and neck	39	7.5
Palmoplantar/Subungual	218	41.8
Upper/Lower limb	47	9.0
Mucosal	118	22.6
Unknown	47	9.0
Histology*		
**LMM**	**3**	**0.6**
**SSM**	**33**	**6.3**
**NM**	**103**	**19.7**
**ALM**	**218**	**41.8**
**MCM**	**118**	**22.6**
**Unclassifiable**	**47**	**9.0**
Ulceration status		
With	234	44.8
Without	123	23.6
Unknown	165	31.6
**Breslow thickness (mm)†**		
**≤ 1**	**27**	**15.0**
**1-4‡**	**80**	**44.4**
**> 4**	**73**	**40.6**
Stage		
I	32	6.1
II	292	55.9
III	131	25.1
IV	67	12.8

* Abbreviation: LMM, lentigo maligna melanoma; ALM, acral lentiginous melanoma; MCM, Mucosal melanoma; NM, nodular melanoma; SSM, superficial spreading melanoma.

† Only includes cutaneous melanoma. MCM and unclassifiable type were excluded.

‡ Includes 4 mm but excludes 1 mm.

### Treatment

#### Surgery

All patients with stage I or II disease had undergone complete resection of their diseases: 95 and 229 cases received local or extended excision, respectively. Among the 131 patients with stage III diseases, local excision, extended excision, and extended excision with regional nodal dissection were performed in 35, 15, and 76 patients, respectively. All patients achieved complete resection except for one patient who had positive margin. In addition, 4 patients did not receive definitive surgery due to patients' preference or poor performance status, including 1 who had palliative surgery.

Breslow thickness were measured and recorded in 180 cases of the cutaneous or mucosal disease in patients with stage I-III diseases after surgery. Measurements of the thickness of the disease were not performed for the rest of the group. Sentinel lymph node dissection was not utilized in the treatment of this group of patients as the majority had non-cutaneous and more advanced diseases.

#### Adjuvant Therapy

Among patients had stage I-III diseases, 155 patients had high-dose IFNα-2b treatment, 109 had adjuvant chemotherapy with or without radiation, and 2 patients received adjuvant radiotherapy only. In addition, 184 patients received no adjuvant therapy.

#### Treatment for Stage IV Disease

All patients with stage IV were treated with systemic treatment using chemotherapy and or targeted therapy (on trial basis). One patient with unknown primary had definitive surgical resection to the solitary metastatic focus, and 62 cases had palliative surgery. The remaining 5 cases had no surgery.

### Clinical staging

Since Breslow thickness was available in 180 of the 455 patients underwent surgery, clinical staging was based on the available knowledge of T-category, regional nodal status, and distant metastasis.

Thirty-two patients presented with limited primary disease (≤ 1.0 mm or ≤ 2.0 mm without ulceration based on pathology study or clinical evaluation) were staged as stage I disease. A total of 292 patients with more advanced T-disease on pathology or physical examination but no evidence of regional or distant metastasis were classified as stage II melanoma. And 131 patients with regional lymph adenopathy without distant metastasis, and 67 patients with distant metastasis were staged as stage III and stage IV diseases, respectively, according to the AJCC clinical classification for melanoma.

Since close to 40% of all patients had stage III or IV diseases, and Breslow thickness were recorded in less than 70% of patients with stage I and II diseases, pathological TNM staging was not established in our database.

### Overall Survival

**Figure 2:**
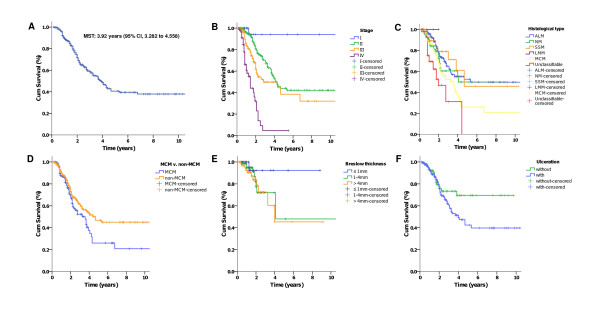
**Kaplan-Meier analyses of overall survival (OS) for the entire group of patients according to different stratums by prognostic factors (overall comparison was administered by Mantel-Cox log-rank test)**. Overall survival (A), OS based on stage (*P *< .001) (B), histology (*P *< .001) (C), MCM and non-MCM patients (*P *= .036) (D), Breslow thickness (*P *= .29) (E), and Ulceration status (*P *= .08) (F).

The 5-year overall survival rate of all 522 patients was 41.6%. The median survival time (MST) was 3.92 years (95% CI, 3.282 to 4.558) (Figure 2A).

**Figure 3:**
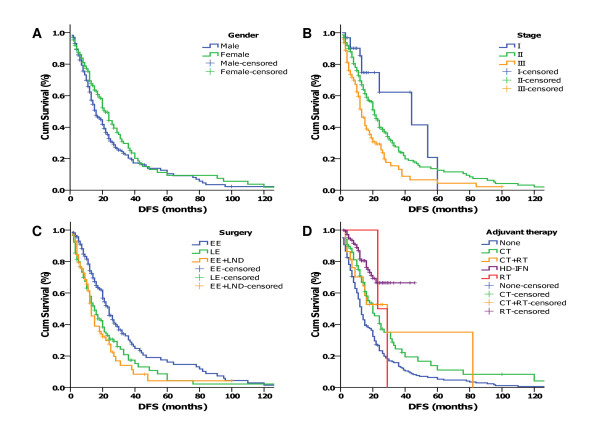
**Kaplan-Meier analyses of disease-free survival (DFS) of 450 patients with stage I-III melanoma received definitive therapy according to different stratums by prognostic factors (overall comparison was performed by Mantel-Cox log-rank test): (A) Male vs. Female (P = .035), (B) DFS estimates according to stage (P < .001), (C) DFS of patients receiving different excision (EE for extended excision, LE for local excision, LND for lymph node dissection) of primary tumor (P < .001), (D) DFS of patients receiving different adjuvant therapy (P < .001)**.

The overall survival rates at 5-years stratified by stages at diagnosis were 94.1%, 44.0%, 38.4%, and 4.6%, respectively for stages I-IV diseases (Figure 2B) (P < 0.001); The median survival for stage I patients was not reached after a median follow-up of 12 months (range: 3-48 months). The median survival time were 4.25 years (95% CI, 3.557-4.943), 2.83 years (95% CI, 0.595-5.065) and 1.42 years (95% CI, 1.166-1.674), respectively for patients with stage II, III, and IV diseases.

The median survival time was 3.58 versus 4.67 years (P = 0.036) for patients with mucosal melanoma (MCM) versus non-MCM (Figure 2D). And the 5-year survival rates for MCM and ALM were 26.8% versus 53.9%, respectively (P = 0.003)

No significant difference in OS was observed among patients with different Breslow thickness of the primary tumor, although the 5-year OS of patients with disease ≤ 1 mm (92.3%) was higher than the other 2 groups (48.9% and 50.1% for disease of 1-4 mm and > 4 mm in thickness) (Figure 2E).

Univariate analyses revealed that histological type, clinical stage, as well as origin of the disease (mucosa vs. non-mucosa) were significant prognosticators for overall survival (Table 2).

**Table 2:** Univariate analyses of prognostic factors for OS in patients with malignant melanoma

Factors	Median OS (y)	P value
Histological type		
ALM	5.330	< 0.001
NM	4.080	
SSM	4.670	
MCM	3.580	
Unclassifiable	2.000	
**MCM vs. non-MCM**		
**MCM**	**3.580**	**0.036**
**Non-MCM**	**4.670**	
Primary location		
Trunk	4.670	< 0.001
H&N	Not reached	
Palmoplantar/Subungual	10.830	
Upper/Lower limb	4.000	
Mucosal	3.580	
Unknown	2.000	
**Stage**		
**I**	**not reached**	**< 0.001**
**II**	**4.250**	
**III**	**2.830**	
**IV**	**1.420**	
Gender		
Male	3.830	0.715
Female	3.920	
**Age**		
**≤ 65 y**	**3.750**	**0.127**
**> 65 y**	**not reached**	
Ulceration		
with	4.000	0.080
without	not reached	
Breslow thickness*		
**≤ 1 mm**		**0.290**
**1-4 mm**	**4.080**	
**> 4 mm**	**4.000**	

*Limited to 180 cases with recorded Breslow thickness

### Disease-free Survival and Prognostic-free Survival

The 5-year disease-free survival (DFS) rate and the median survival time of 455 patients with stage I-III diseases were 12.3% (95% CI, 7.4%-17.2%) and 20 months, respectively.

The histology subtypes, Breslow thickness, presence of ulceration, location of the primary disease, and age had no significant value in predicting DFS in univariate analyses (Table 3).

**Table 3:** Univariate analyses of prognostic factors for DFS in patients with malignant melanoma

Factors	Median DFS (months)	P value
Histological type		
ALM	20.0	0.716
NM	20.0	
SSM	28.0	
LMM	16.0	
MCM	17.0	
Unclassifiable	16.0	
**Primary location**		
**Trunk**	**25.0**	**0.721**
**H&N**	**16.0**	
**Palmoplantar/Subungual**	**20.0**	
**Upper/Lower limb**	**21.0**	
**Mucosal**	**17.0**	
**Unkown**	**16.0**	
Stage		
I	44.0	< 0.001
II	21.0	
III	13.0	
**Gender**		
**Male**	**16.0**	**0.035**
**Female**	**22.0**	
Age		
≤ 65 y	20.0	0.709
> 65 y	20.0	
**Surgery**		
**Local resection**	**15.0**	**< 0.001**
**Extended resection**	**24.0**	
**Ext. resection w/nodal dissection**	**13.0**	
Adjuvant therapy		
None	13.0	< 0.001
Chemotherapy	20.0	
Chemoradiation	29.0	
IFN	Not reached	
Radiation	23.0	
**Ulceration**		
**with**	**20.0**	**0.488**
**without**	**20.0**	
Breslow thickness*		
≤ 1 mm	38.0	0.178
1-4 mm	21.0	
> 4 mm	13.0	

*Limited to 180 cases with recorded Breslow thickness

Univariate analysis of all factors for progression-free survival (PFS) among patients with stage IV diseases was not significant.

### Prognostic Factors

Potential factors for overall survival (OS) of all patients, disease-free survival (DFS) for patients with stage I-III diseases indicated by univariate analyses described above that demonstrated significance or a trend (P < 0.10) were further analyzed in multivariate analysis to identify the independent prognostic factors.

The results of multivariate analysis indicated that stage at diagnosis and the presence of ulceration were two significant predictive factors, whereas anatomic origin of the disease and histological subtype provided no significant prognostic value for OS. Surgical modality (extended surgery vs. local excision) and the use of adjuvant therapy were significant prognosticators for DFS of patients with stage I-III malignant melanoma. Stage of the disease demonstrated a trend in predicting DFS in non-metastatic melanoma patients (Table 4).

**Table 4:** Multivariate analyses of prognostic factors for OS and DFS

Overall Survival		Disease-Free Survival*	
**Prognosticators**	**P Value**	**Prognosticators**	**P Value**

Stage	< 0.001	Stage	0.10

Ulceration	0.043	Surgical Technique	0.004

Histological Type	0.410	Adjuvant Therapy	< 0.001

Anatomic Region	0.251	Gender	0.351

* DFS in non-metastatic patients who received definitive surgery.

## Discussion

In the current analyses of 522 Asian patients diagnosed with malignant melanoma, we discovered that histology subtypes of melanoma diagnosed in Asian patients differ substantially from those reported in Western populations. Specifically, the two most commonly diagnosed subtypes were acral lentiginous melanoma (ALM) and mucosal melanoma (MCM), which accounted for close to 65% of all patients collectively. The overall survival of Asian patients with melanoma was clearly suboptimal: The 5-year overall and disease-free survival (DFS) rates and median survival time were 41.6% and 43 months, and 12.3% and 20 months, respectively. These results were substantially worse than those observed in the SEER data from the United States (5-year survival rate of 91.4%) [12]. However, they were more comparable to those patients treated for high-risk disease at 37%/1.7 years and 26%/1.98 years, respectively for overall survival and DFS [13].

A number of prognostic factors were analyzed, and demonstrated that in addition to the two most commonly observed prognostic factors emphasized by the current version of AJCC staging system, i.e., stage of the disease and the presence of ulceration, other factors including the pathology subtypes and the origin of the primary disease were not significant for predicting overall survival (OS) in multivariate analyses. Nevertheless, the use of adjuvant therapy and extent of surgery were found to be significant factors, and stage at diagnosis showed a clear trend, in predicting the disease-free survival (DFS) after treatment for patients with non-metastatic melanoma.

Stage and the extent of the primary disease (i.e., Breslow thickness) have been repeatedly confirmed to be the most important prognostic indicators for melanoma [3,14,15]. However, most evidences presented in the literatures were originated in endemic regions particularly Western countries, and the most commonly diagnosed subtype of malignant melanoma is superficial spreading melanoma (SMM). Due to the high prevalence of the disease, knowledge on diagnosis and screening is readily available and diagnoses are usually made in relatively earlier stages. The applicability of the AJCC/UICC TNM staging system in melanoma patients from non-endemic regions particularly for histological subtypes rarely observed in endemic regions has not been adequately addressed. Although malignant melanoma is a relatively rare disease in Asia, the incidence of melanoma is increasing. In a nationwide survey of 4495 cases of melanoma from 1992 to 1998 in Japan, the incidence of the disease increased 5-folds [16]. A similar pattern was observed in the metropolitan areas in China, although systemically established tumor registry is lacking.

The lack of knowledge of the disease in Asian patients clearly hampers the understanding thus the development of proper treatment strategy in this particular group of patients, as well as further research for more effective diagnosis and treatment. Therefore, we consider our finding important as the results confirmed the applicability of the updated AJCC staging to Asian patients with different subtypes of malignant melanoma. Both univariate and multivariate analyses of our data showed that clinical staging as well as presence of ulceration were two significant prognosticator for overall survival, in consistent with those reported in endemic regions including United States and Australia, regardless of histological subtypes [10-18]. In addition, a trend was demonstrated in our series for clinical staging in predicting DFS for patients with non-metastatic disease.

Among the five histological types in our series, ALM is the most common type in China accounting for nearly half of all patients, while the sum of NM, SMM and LMM is less than half of ALM. These results are consistent with the data from other Asian countries [7-9], but differed from those reported in the endemic regions where SSM, NM, LMM, and ALM account for > 70%, 15%, 13%, and 2-3%, respectively [3,15,18]. It is suggested that racial status play a key role in worldwide proportion of different histological types. In 1976, RJ Reed first described ALM and noted that this type of melanoma was the most common expression of melanoma in blacks [20]. Lately, ALM was found to be more commonly diagnosed in Asians and African Americans. In addition, African-Americans were found to have significantly shorter survival time as compared to their Caucasian counterparts in the United States [5]. Results from the series from Taiwan also suggested that ALM possess worse prognosis as compared to other types of melanoma such as SSM [9,19,21]. In the current series, we found that histological subtype was a significant prognosticator for overall survival in univariate analysis. However, when stage and presence of ulceration was added, multivariate analyses revealed that histological subtypes per se was not a significant predictor for OS. One of the potential reasons for this discovery was that ALM might have higher incidence of non-cutaneous (e.g., mucosal lesions) thereby a later stage presentation at diagnosis as compared to cutaneous SSM in endemic regions. Whereas in China, stage III and IV diseases were the majority thus the confounding effect of late diagnosis of ALM does not exist. Since the distribution of various subtypes of melanoma in our series was uneven, directly comparison for clinical presentation and outcome between ALM versus SSM, as well as other pathologies were not feasible.

As far as we know, this is the first large-scale observational study for malignant melanoma in Asian patients. Few reports have been published to address the epidemiology in Asia. In an updated survey from Japan published by Ishihara et al, malignant melanoma demonstrated a clear increasing incidence [22]. Furthermore, ALM and nodular melanoma (NM) were the cost common types and collectively accounted for close to 2/3 of all melanoma diagnosed in Japan. These data collide well with our results and those observed in Taiwan [9]. However, evaluation of the effects of certain treatment modality in the Japanese survey such as prophylactic lymph node dissection and adjuvant systemic therapy were inconclusive, and detailed knowledge on treatment as well as outcome is lacking due to the nature of a survey. On the other hand, our data demonstrated a clear benefit of adjuvant therapy, and suggested an efficacy of aggressive treatment in melanoma of Asian patients.

One of the prognostic factors that have not been addressed previously in literature is the mucosal origin of the primary. Mendenhall et al. reported the head and neck mucosal melanoma to be a rare entity comprising less than 1% for all Western melanomas. The likelihood of local recurrence after resection is approximately 50% with 5-year survival rates ranging from 20% to 50% [23]. Since mucosal melanoma occurred in 22.6% of all cases in our series, it was analyzed against non-MCM as a prognostic factor for OS and was found to be significant (P = 0.036). The primary lesions of MCM varied from nasal cavity, paranasal sinuses, oral cavity, choroids, to cervix and even vagina (not shown in this article). The features of occult onset, early recurrence after resection, and present at an advanced stage even with metastasis contribute to the poor survival of MCM.

Although our study represented the largest clinical series and analyses of malignant melanoma in Asia, a number of limitations need to be addressed. Firstly, the database for the current series was prospectively designed and prepared based on the characteristics and factors published in the literature; however, the observational nature of the analysis precluded exhaustive record keeping for all the factors in all cases. As a cancer center specialized in the management of malignant melanoma, referral after surgical resection of the disease to our center is not uncommon. Malignant melanoma is among one of the rarest cancers in China, thus knowledge on proper diagnosis and treatment may not be readily available among the primary care physicians. As such, a number of parameters of the disease including Breslow thickness and the presence of ulceration were not recorded exhaustively. Thickness is the primary determinant of T-category in AJCC staging system of melanoma and in the 6th edition of AJCC in 2002, thickness thresholds were revised to 1.0, 2.0 and 4.0 mm due to a large statistic result of 17,600 patients [10,18]. Although our data revealed the 5-year OS of patients with thickness ≤ 1 mm (92.3%) was substantially higher than those of 1-4 mm (48.9%) and > 4 mm (50.1%), the incomplete data especially the extent of the primary disease may be the key reason that Breslow thickness was insignificant in predicting the overall survival in multivariate analyses. Another potential reason might be the advanced stage at presentation, i.e., the majority of patients had lymph node metastasis and distant metastasis.

Since malignant melanoma is a relatively rare disease in Asia, the majority of patients initiated their treatment in our center were accrued into clinical trials, especially those with more advanced, i.e., stage II-IV, diseases. The effect of the heterogeneous treatment modalities used in clinical trials may also affect the treatment outcome. The results of a recently published prospective randomized trial showed that adjuvant radiation therapy to the regional lymph nodes significantly improved DFS in patients with high-risk melanoma after Lymphadenectomy [24]. In addition, adjuvant systemic treatment with interferon in patients with advanced disease is controversial, based on results of systemic reviews and meta-analyses [25,26]. Furthermore, most of these series were published in endemic regions and ALM and MCM melanoma are usually not a substantial component of the samples studied. Our analyses showed that adjuvant treatments and types of surgery in patients with non-metastatic disease might be a significant prognosticator for DFS. These findings appeared similar to those demonstrated in the literature based on other subtypes of melanoma. However, the selection of type of surgery and adjuvant treatment largely depended on the extent of disease, i.e. clinical staging in our series. The observation of an improved DFS but not OS by more extended surgical subtypes questioned whether improved local control could translate to survival in the more commonly diagnosed melanoma subtypes in Asia. Similar questions also need to be answered for the use of aggressive local and/or systemic adjuvant therapy. Clearly, more effective treatment is clearly needed. Unfortunately, the heterogeneity of our adjuvant treatment strategy precluded detailed and meaningful analyses of the efficacy of individual types of therapy.

Patients with stage IV malignant melanoma are usually treated with systemic biological and/or chemotherapy. However, treatment for metastatic melanoma has always been a challenge, and aggressive treatment including combining chemotherapy and immunotherapy failed to show additive efficacy [27]. Furthermore, targeted therapy such as sorafenib or bevacizumab has been the focus of study for metastatic disease; however, most of the clinical trials revealed moderate or limited efficacy [28-30]. Currently, only one targeted agent demonstrated significant efficacy when used alone [31]. In our institute, stage IV melanoma patients are encouraged to participate clinical trial(s), and most of the 61 patients with stage IV disease were accrued in prospective studies including a combined molecular targeted therapy regimen. The outcomes of these clinical trials are pending.

Although the current series presented the largest clinical data in Asia, epidemiology and etiology of malignant melanoma diagnosed in China were not the focus of the study. One of the interesting issues that await further research is the influence of sunlight to the incidence of cutaneous melanoma (not including ALM) in China. Currently there is no epidemiologic research reported from Asia that has addressed the cause-effect association between sun exposure and cutaneous melanoma. Although the protective effect of melanin in Asian population is well known, the anecdotal observation of the increasing incidence in China may be associated with the lifestyle changes. In addition, as a tertiary cancer institute specialized in melanoma treatment, the number of more advanced disease seen in our center may be overestimated. As such, a regional statistical investigation is needed and is currently under active planning to address the epidemiology of malignant in melanoma more effectively.

## Conclusions

Most malignant melanoma patients in China were diagnosed with locally advanced disease (i.e., stage II or above), and their prognoses were suboptimal. ALM and MCM are the two most commonly diagnosed pathological subtypes of malignant melanoma in China. Clinical staging and presence of ulceration was significantly associated with clinical outcome in terms of OS, while treatment strategy including extent of surgery and use of adjuvant therapy were significant predictors of DFS.

## Competing interests

The authors declare that they have no competing interests.

## Authors' contributions

ZC, SL, and JG participated in the study design. ZC, SL, XS, LS, CC, and MH participated in data collection and analysis. All authors participated in the interpretation and manuscript writing. SL, ZC, and JG participated in editing and proof reading. All authors read and approved the final manuscript.

## Pre-publication history

The pre-publication history for this paper can be accessed here:

http://www.biomedcentral.com/1471-2407/11/85/prepub
